# Identifying influential spreaders by gravity model

**DOI:** 10.1038/s41598-019-44930-9

**Published:** 2019-06-10

**Authors:** Zhe Li, Tao Ren, Xiaoqi Ma, Simiao Liu, Yixin Zhang, Tao Zhou

**Affiliations:** 1 Software College, Northeastern University of China, Shenyang, 110819 P.R. China; 2 School of Science and Technology, Nottingham Trent University, Nottingham, NG11 8NS United Kingdom; 3 CompleX Lab, University of Electronic Science and Technology of China, Chengdu, 611731 P.R. China

**Keywords:** Complex networks, Statistical physics

## Abstract

Identifying influential spreaders in complex networks is crucial in understanding, controlling and accelerating spreading processes for diseases, information, innovations, behaviors, and so on. Inspired by the gravity law, we propose a gravity model that utilizes both neighborhood information and path information to measure a node’s importance in spreading dynamics. In order to reduce the accumulated errors caused by interactions at distance and to lower the computational complexity, a local version of the gravity model is further proposed by introducing a truncation radius. Empirical analyses of the Susceptible-Infected-Recovered (SIR) spreading dynamics on fourteen real networks show that the gravity model and the local gravity model perform very competitively in comparison with well-known state-of-the-art methods. For the local gravity model, the empirical results suggest an approximately linear relation between the optimal truncation radius and the average distance of the network.

## Introduction

Network science is playing an increasingly significant role in many domains including physics, sociology, engineering, biology, management, and so on^1^. The heterogeneous nature of real networks^2^ asks for a crucial question: How to quantitatively measure a node’s importance in a dynamical process? Taking spreading dynamics as an example, a popular star in Twitter may remarkably accelerate a rumor and a few superspreaders could largely expand the epidemic prevalence of a disease^3^. Therefore, a good answer to the above question, namely an efficient algorithm to identify influential spreaders in complex networks, can help to better control the outbreak of an epidemic^4^, optimize the use of limited resources to facilitate the dissemination of information^5^, prevent catastrophic disruptions of power grid or the Internet^6^, discover the candidates of drug target and essential proteins^7^, and so on. Till far, most known methods only make use of the structural information^8^, which can be roughly classified into neighborhood-based centralities and path-based centralities.

Typical representatives of the neighborhood-based centralities are degree centrality^9^ (DC), H-index^10^ and *k*-shell decomposition method^11^ (KS). For DC, nodes with larger degrees are more influential. For H-index, nodes connecting with many large-degree neighbors are more influential. KS assigns a *k*-shell index to each node based on its topological location, where nodes closer to the core of the network will get higher *k*-shell indices, and nodes in the periphery will get lower *k*-shell indices. The nodes with higher *k*-shell indices are considered to be more influential. Besides, PageRank^12^ and LeaderRank^13^ are two representative neighborhood-based iterative methods, both suggesting that the influence of a node is determined by the influences of its neighbors. Two well-studied path-based centralities are closeness centrality^14^ (CC) and betweenness centrality^15^ (BC). CC claims that a node averagely closer to other nodes is more influential while BC assumes that a node locating in many shortest paths is of high influence.

Inspired by the gravity law, recently, Ma *et al*.^16^ proposed two gravity-law-based algorithms by considering both neighborhood information and path information (see Methods for the details of algorithms). Analogously, we proposed a variant algorithm named gravity model (GM), which also takes into account both neighborhood information and path information, where a node with larger degrees (neighborhood information) and averagely shorter distances to other nodes (path information) is more influential. Furthermore, we propose a local version of the gravity model (named as local gravity model, LGM for short) to lower the computational complexity and reduce the possible noise caused by interactions at distance. Such local model only accounts for pairwise interactions within a truncation radius. Empirical results show that GM and LGM perform very competitively in comparison with well-known state-of-the-art methods. In particular, for LGM, an empirically linear relation between the optimal truncation radius and the average distance of the network is observed.

## Results

### Algorithms

Individually speaking, nodes with large degrees are likely to be more influential. In addition, a node is of higher impacts on nearby nodes^17^. According to the above issues and inspired by the gravity law, we regard the degree of a node as its mass, and the shortest distance between two nodes as their distance. Hence a node *i*’s influence can be estimated as

1 $$S(i)=\sum _{j\ne i}\,\frac{{k}_{i}{k}_{j}}{{d}_{ij}^{2}},$$

where *k*_*i*_ is the degree of node *i*, *d*_*ij*_ is the shortest distance between node *i* and node *j*, and *j* runs over all nodes other than *i*. Obviously, a node with many neighbors and be close to most nodes is more influential according to Eq. 1. Such method is named as gravity model as it adopts the formula of the gravity law.

Although GM can identify the nodes averagely closer to other nodes and with larger degrees, it has two shortcomings. Firstly, to calculate shortest distances between all node pairs is time-consuming for large-scale networks^18^. Secondly, in real propagation a node is hard to impact other nodes at distance and to estimate the interacting strength between distant nodes is usually inaccurate since the step-by-step decaying influence will be disturbed by accumulated noise^19^. Therefore, by introducing a truncation radius, we only consider the pairwise interactions within the truncation radius. Hence a node *i*’s influence can be estimated as

2 $${S}_{R}(i)=\sum _{{d}_{ij}\le R,j\ne i}\,\frac{{k}_{i}{k}_{j}}{{d}_{ij}^{2}},$$

where *R* is the truncation radius. Such method (Eq. 2) is named as local gravity model as it only takes into account local information of the network.

### Data description

In this paper, fourteen real networks from disparate fields are used to test the performance of GM and LGM, including three collaboration networks (Jazz, NS and GrQc), four communication networks (EEC, Email, PG and Enron), four social networks (PB, Facebook, WV and Sex), one transportation network (USAir), one infrastructure network (Power) and one technological network (Router). Jazz^20^ is a collaboration network of jazz musicians. NS^21^ is a co-authorship network of scientists working on network science. GrQc^22^ is a collaboration network of eprint articles in arXiv categories General Relativity and Quantum Cosmology. EEC^23^ describes email interchanges between institution members of a large European research institution. Email^24^ describes email interchanges between users including faculty, researchers, technicians, managers, administrators, and graduate students of the Rovira i Virgili University. PG^22^ is a snapshot of the Gnutella peer-to-peer file sharing network from August 2002. Enron^25^ is the Enron email network. PB^26^ is a network of US political blogs. Facebook^27^ describes social circles from Facebook. WV^28^ is a network of Wikipedia who-votes-on-whom. Sex^29^ is a bipartite network in which nodes are females (sex sellers) and males (sex buyers) and links between them are established when males write posts indicating sexual encounters with females. USAir^30^ is the US air transportation network. Power^31^ is the power grid of the western United States. Router^32^ is a symmetrized snapshot of the structure of the Internet at the level of autonomous systems. These networks’ topological features (including the number of nodes, the number of links, the average degree, the average distance, the clustering coefficient^31^, the assortative coefficient^33^, the degree heterogeneity^34^ and the epidemic threshold^35^ of the SIR model^36^) are shown in Table 1.

**Table 1:** The basic topological features of the fourteen real networks.

Networks	*N*	*E*	〈*k*〉	〈*d*〉	*C*	*r*	*H*	*β*_*c*_
Jazz	198	2472	27.6970	2.2350	0.6334	0.0202	1.3951	0.0266
NS	379	914	4.8232	6.0419	0.7981	−0.0817	1.6630	0.1424
GrQc	4158	13422	6.4560	6.0494	0.6648	0.6392	2.7852	0.0589
EEC	986	16064	32.5842	2.5869	0.4505	−0.0257	2.2912	0.0136
Email	1133	5451	9.6222	3.6060	0.2540	0.0782	1.9421	0.0565
PG	6299	20776	6.5966	4.6430	0.0150	0.0355	2.6764	0.0600
Enron	33696	180811	10.7319	4.0252	0.7081	−0.1165	13.2655	0.0071
PB	1222	16714	27.3552	2.7375	0.3600	−0.2213	2.9707	0.0125
Facebook	4039	88234	43.6910	3.6925	0.6170	0.0636	2.4392	0.0095
WV	7066	100736	28.5129	3.2475	0.2090	−0.0833	5.0992	0.0069
Sex	15810	38540	4.8754	5.7846	0	−0.1145	5.8276	0.0365
USAir	332	2126	12.8072	2.7381	0.7494	−0.2079	3.4639	0.0231
Power	4941	6594	2.6691	18.9892	0.1065	0.0035	1.4504	0.3483
Router	5022	6258	2.4922	6.4488	0.0329	−0.1384	5.5031	0.0786

*N* and *E* are the number of nodes and links. 〈*k*〉 and 〈*d*〉 are the average degree and the average distance. *C* and *r* are the clustering coefficient and the assortative coefficient. *H* is the degree heterogeneity. *β*_*c*_ is the epidemic threshold of the SIR model.

### Empirical results

We apply the well-known SIR model^36^ to compare the rankings of influences produced by algorithms and simulations. Initially, one node (called seed) in the network is in the infected state (I) and the others are in the susceptible state (S). Each of the infected nodes can infect its susceptible neighbors with probability *β*. And in each step, every infected node changes to be recovered and will never participate in the dynamics with probability *λ*. The spreading process repeats until there are no more infected nodes in the network. The influence of any node *i* can be estimated by

3 $$F(i)={N}_{r}/N,$$

where *N*_*r*_ is the number of recovered nodes at the end of the dynamics. For simplicity, we set *λ* = 1, and the corresponding epidemic threshold^34^ is

4 $${\beta }_{c}\approx \frac{\langle k\rangle }{\langle {k}^{2}\rangle -\langle k\rangle },$$

where 〈*k*〉 and 〈*k*^2^〉 denote the average degree and the second-order moment of the degree distribution.

**Figure 1:**
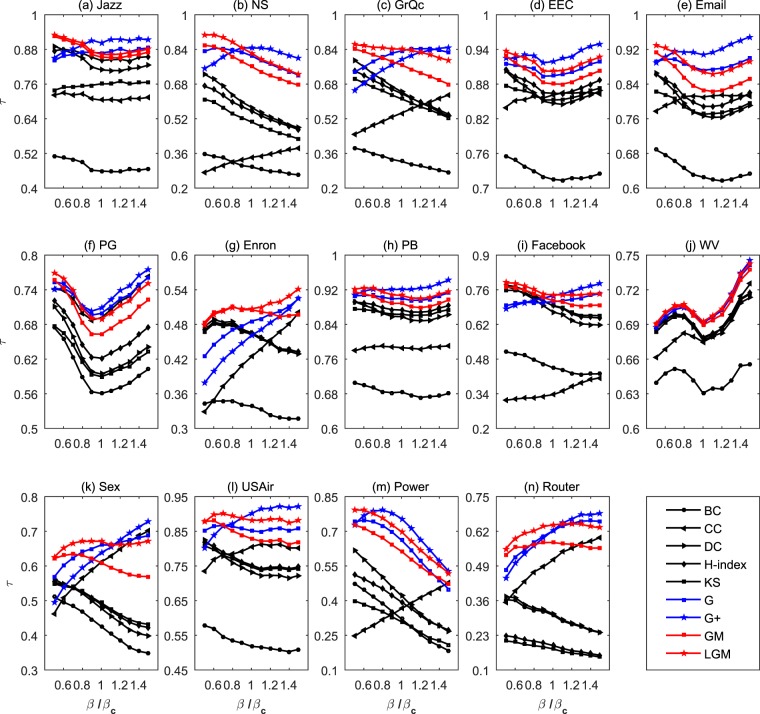
The algorithms’ accuracies for different *β*, measured by the Kendall’s Tau (*τ*).

Given a network and the transmission probability *β*, to obtain the standard ranking of nodes’ influences, we implement 1000 independent runs, in each run every node is selected once as the seed once. The accuracy of an algorithm is measured by the Kendall’s Tau (*τ*)^37^ between the standard ranking and the ranking by the algorithm (see details in Methods). A larger value of *τ* means a stronger correlation between the two sequences and thus a better performance. Table 2 compares the accuracies of the two proposed algorithms (i.e., GM and LGM) and seven benchmark algorithms (see details about the benchmark algorithms in Methods). The transmission probability for each case is fixed as *β* = *β*_*c*_ (for more values of *β*, see Fig. 1) and the parameters in relevant algorithms are all adjusted to their optimal values subject to the largest *τ*.

**Table 2:** The algorithms’ accuracies for *β* = *β*_*c*_, measured by the Kendall’s Tau (*τ*).

Networks	BC	CC	DC	H-index	KS	G	G+	GM	LGM
Jazz	0.4590	0.7043	0.8088	0.8417	0.7608	0.8677	**0**.**9025**	0.8533	0.8634
NS	0.2979	0.3415	0.5728	0.5561	0.5051	0.8110	**0**.**8464**	0.7611	0.8231
GrQc	0.3231	0.5464	0.6443	0.6362	0.6115	0.8337	0.7922	0.7684	**0**.**8417**
EEC	0.7151	0.8610	0.8468	0.8641	0.8525	0.8943	**0**.**9189**	0.8803	0.9022
Email	0.6254	0.8104	0.7665	0.7887	0.7707	0.8720	**0**.**9076**	0.8265	0.8671
PG	0.5605	0.6916	0.5941	0.6216	0.5897	0.6992	**0**.**7082**	0.6632	0.6900
Enron	0.3387	0.4241	0.4657	0.4654	0.4636	0.4859	0.4610	0.5055	**0**.**5075**
PB	0.6839	0.7865	0.8580	0.8732	0.8633	0.9001	**0**.**9211**	0.8887	0.9067
Facebook	0.4450	0.3362	0.6704	0.6948	0.6965	0.7117	0.7361	0.7160	**0**.**7394**
WV	0.6305	0.6748	0.6763	0.6788	0.6778	0.6919	0.6917	0.6895	**0**.**6926**
Sex	0.4251	0.6119	0.4774	0.4889	0.4934	0.6606	0.6386	0.6092	**0**.**6713**
USAir	0.5181	0.8052	0.7320	0.7525	0.7470	0.8514	**0**.**9012**	0.8286	0.8817
Power	0.3205	0.3653	0.4207	0.3935	0.3084	0.6610	**0**.**7544**	0.6128	0.6947
Router	0.3059	0.5120	0.3107	0.1917	0.1791	0.6216	0.6226	0.5782	**0**.**6441**

The best performed algorithm for each network is emphasized by bold.

As shown in Table 2, both GM and LGM are very competitive. In particular, G+ and LGM perform best among the nine algorithms. Notice that, G+ also adopts the gravity formula^16^ (see Methods) but a node’s mass in G+ is defined as its *k*-shell index so G+ is indeed a global index. The results reported in Table 2 demonstrate the advantage of gravity models (e.g., G, G+, GM, LGM) and show that a local index (LGM) can outperform most benchmark algorithms including some global indices. As shown in Fig. 1, results for other values of *β* not too far from the threshold are consistent to the one at *β*_*c*_, suggesting the robustness of our findings.

**Figure 2:**
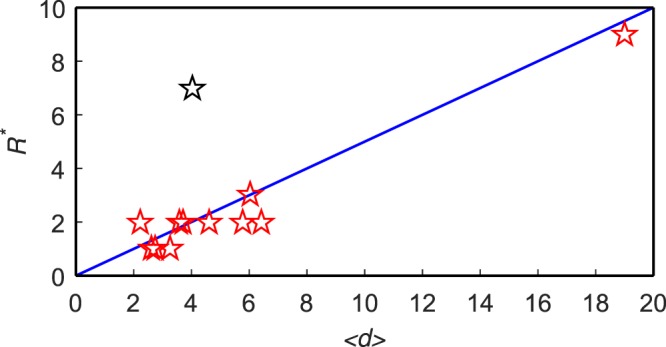
The relation between R^*^ and 〈*d*〉 for *β* = *β*_*c*_. Fourteen pentagrams represent fourteen networks and the slope of the blue line is 1/2. The pentagram in black is the outlier – the Enron network. Although the optimal truncation radius R^*^ = 7 is much different from what Eq. 5 predicts (i.e., *R* = 2), the algorithmic accuracy at *R* = 2 (*τ* = 0.4949) is very close to the best accuracy at R^*^ = 7 (*τ* = 0.5075) in comparison with the traditional methods (e.g., about 0.34 for BC, 0.42 for CC and 0.46 for DC, KS and H-index). That is to say, to apply Eq. 5 can still achieve much better algorithmic performance than the traditional methods.

Since to determine the optimal truncation radius, denoted by R^*^, asks for more computation, we want to see whether topological information can be used to profile R^*^. As shown in Fig. 2, R^*^ approximately scales linearly with the average distance, as

5 $${R}^{\ast }\approx \frac{1}{2}\langle d\rangle $$

at *β* = *β*_*c*_. Such approximately linear relation also holds for other values of *β* not so far from *β*_*c*_. This empirical relation can save computational cost in practice.

## Discussion

To measure influences of nodes in a certain networked dynamics, a straightforward method is to estimate the interacting strengths between node pairs in advance. The gravity law is a simple, elegant and representative formula that estimates the interacting strength between two nodes by simultaneously considering the intrinsic influences of the two nodes themselves and the distance between them. In this paper, the gravity model (Eq. 1) makes use of both the neighborhood information and the path information, which were separately adopted in many previous methods. Furthermore, to reduce the computational complexity and to avoid the accumulated noises through long paths, we proposed a local version of the gravity model (LGM, see Eq. 2). Both GM and LGM are very competitive, and of particular interests, the LGM requires less computation yet performs even better. Indeed, LGM is one of the two best-performed methods among many well-known benchmark algorithms.

A potential disadvantage of LGM is that it has a free parameter, namely the truncation radius *R*. The negative effects of the existence of *R* are twofold. Firstly, it asks for more computation to determine the optimal value of *R*. Secondly, if the optimal value, say R^*^, is very large, the computational complexity of LGM will be more or less the same to GM. Fortunately, as shown in Fig. 2, we found an empirical relation between R^*^ and the average distance 〈*d*〉, so that if the computational resource is highly limited, we can use the relation (see Eq. 5) to approximate R^*^. In addition, since most real networks are of small-world property^31,38^, R^*^ should be small and thus it requires much less computation than GM. Fortunately, the difference between two rankings of nodes produced by neighboring *R* will quickly converge to a very small value, so that to choose a small value of *R* will probably perform very well. In Table 3, we show the values of *τ*(*R*), which is the Kendall’s tau between two rankings of nodes’ influences with truncation radius being *R* and *R* + 1. One can observe that after *R* = 5, all networks are of *τ*(*R*) > 0.97 and a half of them are of *τ*(*R*) > 0.99. This indicates a strong saturation, namely the increasing of *R* will produce almost the same rankings if the value of *R* is already large.

**Table 3:** The Kendall’s Tau between two rankings of nodes’ influences produced by the LGM with truncation radius *R* and *R* + 1.

Networks	*R* = 1	*R* = 2	*R* = 3	*R* = 4	*R* = 5
Jazz	0.9748	0.9927	0.9976	0.9981	0.9993
NS	0.9348	0.9629	0.9752	0.9797	0.9829
GrQc	0.9197	0.9161	0.9380	0.9628	0.9721
EEC	0.9773	0.9882	0.9963	0.9978	0.9988
Email	0.9596	0.9770	0.9840	0.9927	0.9963
PG	0.9413	0.9596	0.9766	0.9886	0.9957
Enron	0.8479	0.8958	0.9274	0.9611	0.9793
PB	0.9682	0.9865	0.9956	0.9977	0.9984
Facebook	0.8797	0.9431	0.9768	0.9842	0.9899
WV	0.9668	0.9760	0.9958	0.9982	0.9989
Sex	0.9039	0.9042	0.9500	0.9615	0.9712
USAir	0.9607	0.9697	0.9858	0.9912	0.9939
Power	0.9486	0.9672	0.9717	0.9754	0.9785
Router	0.8416	0.9007	0.9402	0.9600	0.9720

Another similar model (named G+, see Eq. 11) shows very close performance to LGM. In comparison, LGM is more efficient since it completely depends on the local topological structure and thus can be calculated not only faster but also under the case where the global topology is not known. In the absence of global topology, G+ cannot be obtained since it sets a node’s *k*-shell index as its mass, and to determine the *k*-shell index needs the knowledge of the whole network. In despite the difference between G+ and LGM, the very good performance of G+ and LGM strongly suggest the validity and advantage of the usage of the gravity law to estimate the interacting strength. Of course, both G+ and LGM are very simple and general, which can be further improved by the following aspects (also leaving as open issues for future studies). Firstly, by introducing a few tunable parameters that can adjust the relative importance of mass and distance (e.g., to replace *d*^2^ by some *d*^*a*^ and/or to replace *k* by some *k*^*b*^) may result in more accurate predictions as indicated by known variants of the gravity law in other applications^39^. Secondly, we should explore how the topological features and dynamical processes affect the prediction accuracy and thus improve the original methods by introducing some topology-dependent and/or dynamics-sensitivity items^40,41^. Thirdly, the original gravity law is symmetric, while due to the different roles of different nodes or the essentially asymmetric nature of the dynamics^42,43^, the influence from node *i* onto node *j* could be different from the influence from node *j* onto node *i*, where an asymmetric form of the gravity law may be relevant.

## Methods

### The Kendall’s Tau

The Kendall’s Tau^37^ is an index measuring the correlation strength between two sequences. Considering two sequences with *N* elements, *X* = (*x*_1_, *x*_2_, …, *x*_*N*_) and *Y* = (*y*_1_, *y*_2_, …, *y*_*N*_). Any pair of two-tuples (*x*_1_, *y*_1_) and (*x*_*j*_, *y*_*j*_) (*i* ≠ *j*) are concordant if both *x*_*i*_ > *x*_*j*_ and *y*_*i*_ > *y*_*j*_ or both *x*_*i*_ < *x*_*j*_ and *y*_*i*_ < *y*_*j*_. They are discordant if *x*_*i*_ > *x*_*j*_ and *y*_*i*_ < *y*_*j*_ or *x*_*i*_ < *x*_*j*_ and *y*_*i*_ > *y*_*j*_. If *x*_*i*_ = *x*_*j*_ or *y*_*i*_ = *y*_*j*_, the pair is neither concordant nor discordant. The Kendall’s Tau of two sequences *X* and *Y* can be calculated as

6 $$\tau =\frac{\mathrm{2(}{n}_{+}-{n}_{-})}{N(N-\mathrm{1)}},$$

where *n*_+_ and *n*_−_ denote the number of concordant and discordant pairs, respectively. It can be seen that the extent to which *τ* exceeds zero indicates the strength of the correlation.

### Benchmark centralities

Degree Centrality^9^ of node *i* is defined as

7 $$DC(i)=\sum _{j}\,{a}_{ij},$$

where *A* = {*a*_*ij*_} is the adjacency matrix, that is, *a*_*ij*_ = 1 if *i* and *j* are connected and 0 otherwise.

H-index^10^ of node *i*, denoted by *H*(*i*), is defined as the maximal integer satisfying that there are at least *H*(*i*) neighbors of node *i* whose degrees are all no less than *H*(*i*). Such index is an extension of the famous H-index in scientific evaluation^44^ to network analysis.

Closeness Centrality^14^ of node *i* is defined as

8 $$CC(i)=\frac{N-1}{\sum _{j\ne i}\,{d}_{ij}}\mathrm{.}$$

Betweenness Centrality^15^ of node *i* is defined as

9 $$BC(i)=\sum _{s\ne i,s\ne t,i\ne t}\,\frac{{g}_{st}(i)}{{g}_{st}},$$

where *g*_*st*_ is the number of shortest paths between nodes *s* and *t*, and *g*_*st*_(*i*) is the number of shortest paths between nodes *s* and *t* that pass through node *i*.

Gravity Centrality^16^ (G) of node *i* is defined as

10 $$G(i)=\sum _{j\in {\psi }_{i}}\,\frac{{k}_{s}(i){k}_{s}(j)}{{d}_{ij}^{2}},$$

where *k*_*s*_(*i*) is the *k*-shell index of node *i*, and *ψ*_*i*_ is the set of nodes whose distance to node *i* is less than or equal to 3.

Extended Gravity Centrality^16^ (G+) of node *i* is defined as

11 $${G}_{+}(i)=\sum _{j\in {{\rm{\Lambda }}}_{i}}\,G(j),$$

where Λ_*i*_ is the set of neighbors of node *i*.

## Data Availability

All relevant data are available at https://github.com/MLIF/Network-Data.
